# Extracellular Vesicles Influence the Growth and Adhesion of *Staphylococcus epidermidis* Under Antimicrobial Selective Pressure

**DOI:** 10.3389/fmicb.2020.01132

**Published:** 2020-07-02

**Authors:** Magdalena Zaborowska, Carles Taulé Flores, Forugh Vazirisani, Furqan A. Shah, Peter Thomsen, Margarita Trobos

**Affiliations:** ^1^Department of Biomaterials, Institute of Clinical Sciences, Sahlgrenska Academy, University of Gothenburg, Gothenburg, Sweden; ^2^Centre for Antibiotic Resistance Research (CARe), University of Gothenburg, Gothenburg, Sweden

**Keywords:** implant-associated infections, extracellular vesicles, antimicrobial tolerance, staphylococci, biofilm

## Abstract

*Staphylococcus epidermidis* causes infections associated with orthopedic implants due to its ability to establish persistent biofilms, making infections chronic and hard to treat. Extracellular vesicles (EVs) are part of the bacterial communication system, but the role of *S. epidermidis*-derived EVs in biofilm formation processes and survival is completely unknown. The aims of this study were (i) to investigate the effect of subinhibitory concentrations of antibiotics on vesiculation in *S. epidermidis* and evaluate the role of EVs in bacterial survival and adhesion under antimicrobial selective pressure and (ii) to evaluate whether EVs derived from a gentamicin-resistant *S. epidermidis* strain influence the susceptibility and adhesion of a gentamicin-susceptible strain. A gentamicin-susceptible (GEN^*S*^) strain isolated from implant-associated osteomyelitis was cultured with EVs previously isolated from the same strain growing with subinhibitory concentrations of GEN (0, 0.03, and 0.06 μg × mL^–1^) or with EVs from a gentamicin-resistant (GEN^*R*^) strain. EVs were characterized regarding their size, number and protein content. The growth of *S. epidermidis* cultured with increasing concentrations of GEN (<=> MIC of 0.12 μg × mL^–1^) was recorded, viability was determined by quantitative culturing and fluorescence staining, and biofilm biomass on polystyrene was quantified by crystal violet staining. Cells grown in subinhibitory concentrations of GEN produced a larger number of EVs of similar size but with greater protein content than cells grown in control (Ctrl) conditions (0 GEN). Under antimicrobial pressure, EVs promoted different mechanisms of antimicrobial tolerance depending on the EV and GEN concentrations. Cell adhesion to polystyrene decreased in the presence of 0 and 0.03 μg × mL^–1^ GEN upon EV stimulation. Compared with Ctrl cells, cells treated with EVs from a GEN^*R*^ strain showed increased cell division during the exponential growth phase, faster maximal growth rate, shorter doubling time (8–33 min), and dramatically inhibited cell adhesion. These findings suggest that vesiculation in *S. epidermidis* is a survival response to subinhibitory concentrations of gentamicin. EVs may contribute to bacterial survival through their involvement (1) in the modulation of the growth rate, affecting cell division, and (2) in cell adhesion, decreasing cell attachment to polystyrene and glass.

## Introduction

Biomaterial-associated infection is one of the most frequent complications associated with orthopedic implants and involves a complex interaction between the causative bacteria, the biomaterial, and the host immune response (Arciola et al., 2018). Staphylococci, mainly *Staphylococcus aureus* and *Staphylococcus epidermidis*, are the most common bacteria responsible for these infections and are often difficult to treat due to their ability to form persistent biofilms (Costerton et al., 1999; Darouiche, 2001; Donlan, 2001; Fux et al., 2003; Lewis, 2007; Esteban et al., 2010; Montanaro et al., 2011; Arciola et al., 2012, 2018). Long-term antimicrobial treatment of implant-associated infections is often necessary; in some cases, the infections are not resolved, and the implant needs to be removed. Microorganisms growing in biofilms display significantly increased tolerance to antibiotics (Zaborowska et al., 2017). Among the mechanisms involved in the increased antimicrobial resistance of biofilms are restricted penetration of antimicrobial agents through the biofilm matrix, differential physiological activity of the bacteria in the biofilm, and the presence of a subpopulation of dormant cells in a non-dividing state (Francolini and Donelli, 2010; Ciofu et al., 2017).

Extracellular vesicles (EVs) have been observed for several gram-positive bacterial species, including *S. aureus*, and contain a range of cargo molecules, such as nucleic acids, proteins, lipids, viruses, enzymes, and toxins (Mashburn-Warren and Whiteley, 2006; Lee et al., 2009; Hong et al., 2011; Liu et al., 2018). EV release in gram-positive bacteria has been suggested to be an active metabolic process, with specific sorting mechanisms that determine the contents of EVs. In addition, different environmental conditions have been shown to influence the rate of vesicle production by bacteria (Maredia et al., 2012; Gamalier et al., 2017). Various roles of EVs have been suggested in bacterial physiology and ecology (material exchange, survival and competition) and in microbe-host interactions (infection and invasion, immune evasion, and immune modulation) (Kuehn and Kesty, 2005; Mashburn and Whiteley, 2005; Schooling and Beveridge, 2006; Schooling et al., 2009; Liu et al., 2018). Research into EVs from gram-positive bacteria is a relativity new field; however, multiple studies of outer membrane vesicles (OMVs) derived from gram-negative bacteria have been performed. OMVs are involved in a variety of biological processes, such as cellular defense (Manning and Kuehn, 2011), cell communication (Mashburn and Whiteley, 2005), DNA transfer (Kolling and Matthews, 1999; Yaron et al., 2000; Kulp and Kuehn, 2010; Pérez-Cruz et al., 2013), the pathogenesis and delivery of virulence factors (Kadurugamuwa and Beveridge, 1995; Kolling and Matthews, 1999; Horstman and Kuehn, 2000; Rivera et al., 2010), and the inactivation of antimicrobials by enzymatic degradation (Ciofu et al., 2000). OMVs from gram-negative bacteria have been found in the extracellular matrix of biofilms (Schooling and Beveridge, 2006). These OMVs are involved in mediating interactions within biofilms and protecting bacteria within the biofilm by binding or inactivating harmful molecules such as antibiotics and complement molecules (Kamaguchi et al., 2003; Kulp and Kuehn, 2010). Studies on EVs derived from *S. aureus* showed that they contain the β-lactamase protein BlaZ, which confers penicillin resistance (Lee et al., 2009, 2013). Vesicle production in *S. epidermidis* and the functional roles that these EVs play in interbacterial communication, specifically in bacterial survival and biofilm formation, are unknown.

The aims of this study were (1) to investigate the effect of subinhibitory concentrations of gentamicin on the formation and secretion of EVs in a clinical *S. epidermidis* strain and to evaluate the effect of EVs on the growth and adhesion abilities of the strain when recultured under the same antimicrobial pressure and (2) to examine whether donor EVs isolated from a biofilm-producing/GEN^*R*^ *S. epidermidis* strain promote antimicrobial tolerance and biofilm formation in a recipient clinical *S. epidermidis* strain (non-biofilm-producing/GEN^*S*^).

## Materials and Methods

### Bacterial Strains

Two *S. epidermidis* strains were used in this study: the reference strain ATCC 35984 (strong biofilm-producing; biofilm^*pos*^) and one clinical strain CCUG 64523 (non-biofilm-producing; biofilm^*neg*^), which was isolated from a patient with implant-related osteomyelitis and has been characterized previously (Zaborowska et al., 2017). The study protocol was approved by the Regional Ethics Review Board of Gothenburg (Dnr. 434-09). Susceptibility testing using the *E*-test (bioMérieux, Marcy-l′Étoile, France) was performed; according to the Clinical and Laboratory Standard Institute (CLSI) breakpoints, *S. epidermidis* CCUG 64523 is a gentamicin-susceptible (GEN^*S*^) strain (MIC = 0.094 μg × mL^–1^), and *S. epidermidis* ATCC 35984 is a GEN^*R*^ strain (MIC = 16 μg × mL^–1^) (Clinical and Laboratory Standards Institute [CLSI], 2015). Several control (Ctrl) strains were used – *S. aureus* ATCC 29213 as a quality control for MIC testing and *S. epidermidis* ATCC 35984 and ATCC 12228 as positive and negative Ctrls, respectively – for the microtiter plate assay.

### Vesiculation Under Subinhibitory Concentrations of GEN

#### Isolation of EVs

**FIGURE 1:**
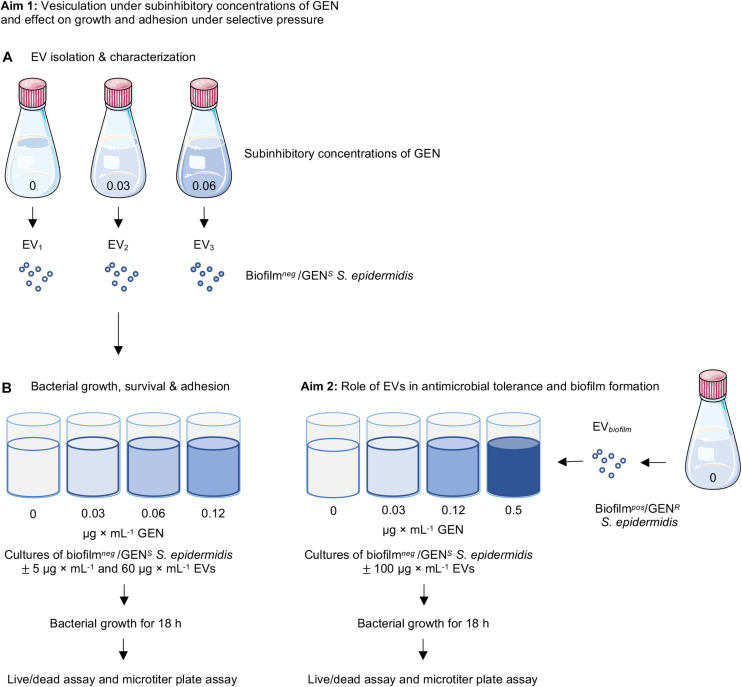
Schematic figure of the experimental system. **(A)** EVs were isolated from non-biofilm-producing *Staphylococcus epidermidis* CCUG 64523 under different culture conditions with and without supplementation of gentamicin (0, 0.03, and 0.06 μg × mL^–1^). Kinetic growth curves of *S. epidermidis* CCUG 64523 with and without gentamicin supplementation (0, 0.03, 0.06, and 0.12 μg × mL^–1^) and with and without stimulation of the three EV types isolated (EV_1_, EV_2_, and EV_3_) were generated. Live/dead fluorescence and microtiter plate assays were performed after 18 h of culture to analyze the effect of EVs on planktonic growth and adherence of bacterial cells to tissue culture plates. **(B)** EVs isolated from the biofilm-producing, gentamycin-resistant strain *S. epidermidis* ATCC35984 were added to cultures of *S. epidermidis* CCUG 64523 with and without gentamicin supplementation (0, 0.03, 0.12, and 0.5 μg × mL^–1^), and kinetic growth curves were generated. Live/dead and microtiter plate assays were performed after 18 h of culture to analyze the effect of EVs on planktonic viability and the adherence of bacterial cells to tissue culture plates.

The methodological format for this study is summarized in Figure 1. The vesiculation of the clinical strain (CCUG 64523) under subinhibitory culture conditions was evaluated as follows (Figure 1A). The strain was cultured overnight on 5% horse blood Columbia agar plates (Media Department, Clinical Microbiology Laboratory, Sahlgrenska University Hospital, Sweden). One colony was cultured in 100 mL of Mueller-Hinton broth (MHB) (Scharlau, Barcelona, Spain) with and without GEN (0, 0.03, and 0.06 μg × mL^–1^) and incubated at 37°C for 22 h with gentle shaking (125 rpm). EVs isolated from cultures supplemented with 0, 0.03, and 0.06 μg × mL^–1^ GEN are hereafter referred to as EV_1_, EV_2_, and EV_3_, respectively. Bacterial cells were removed from the cultures by centrifugation at 3 000 × *g* for 20 min at 4°C. The culture supernatants were sequentially filtered through 0.45- and 0.22-μm pore size vacuum filters (Sarstedt, Nümbrecht, Germany) to remove the remaining bacterial cells. A sterility check was performed by culturing 100 μL of supernatant on blood agar plates. The filtered solution was ultracentrifuged for 20 min at 16 500 × *g* and 4°C in a T-647.5 rotor (Sorvall wx Ultra series, Thermo Scientific, United States) and then filtered through a 0.2 μm mesh to remove any bacterial cell debris. Thereafter, the solution was ultracentrifuged at 150 000 × *g* for 3 h at 4°C. The resulting pellet containing the EVs was washed in PBS, ultracentrifuged again at 150 000 × *g* for 3 h at 4°C, and resuspended in PBS. Three separate batches of EVs were isolated per culture condition (*n* = 3).

#### Characterization of EVs

##### EV number and size: nanoparticle tracking analysis

Nanoparticle concentrations and size distribution profiles of the isolated EVs (EV_1_, EV_2_, and EV_3_) were obtained by nanoparticle tracking analysis (NTA) using the NanoSight LM10/LM14 instrument (NanoSight Ltd., Amesbury, United Kingdom) with NanoSight particle tracking software 3.1. EVs were serially diluted in PBS, injected into the LM14 module, and captured three times for 60 s. The mean and standard error of the mean were calculated from the three captures in triplicate.

##### Protein content: nanophotometry

The total protein concentration of the EVs (EV_1_, EV_2_, and EV_3_) was quantified using a Pierce^®^ BCA Protein Assay kit (Thermo Scientific, Rockford, IL, United States) following the manufacturer’s instructions. The EVs were stored at −80°C until further use.

##### EV morphology: scanning electron microscopy

The detection and morphology of the isolated EVs were evaluated by scanning electron microscopy (SEM): a 10 μL volume of EVs (0.5–1 μg × mL^–1^) in PBS was applied onto 200 mesh Cu 01700-F formvar carbon-coated grids (Ted Pella Inc., Redding, CA, United States). Samples were rinsed after 1 h, fixed in 2% paraformaldehyde for 10 min, rinsed in PBS, postfixed in 2.5% glutaraldehyde, rinsed in dH_2_O, and incubated in 2% uranyl acetate for 15 min. Then, samples were dried, sputter-coated with Au (≈10 nm) and visualized using SEM (Ultra 55 FEG SEM, Leo Electron Microscopy Ltd., United Kingdom) in secondary electron mode, at 5 kV accelerating voltage and 10 mm working distance.

#### Effect of the EVs on Bacterial Growth, Survival, and Adhesion Under Antimicrobial Selective Pressure

##### Growth curves: kinetic optical density measurements

Isolated colonies from overnight blood agar cultures of *S. epidermidis* CCUG 64523 were suspended in 4 mL of MHB until an optical density (OD_546__nm_) of 0.25, equivalent to 10^8^ colony forming units (CFU) × mL^–1^, was reached. A total volume of 200 μL was added to each well, consisting of 100 μL of bacterial suspension (10^5^ CFU × mL^–1^ final well concentration), 50 μL of EVs from *S. epidermidis* CCUG 64523 previously isolated under the selective pressure of different concentrations of GEN (EV_1_, EV_2_, and EV_3_) (final well concentrations of 0, 5, and 60 μg × mL^–1^) and 50 μL of three different concentrations of GEN (0, 0.03, 0.06, and 0.12 μg × mL^–1^ final well concentration) in Nunc 96-well plates (Thermo Fisher Scientific, Roskilde, Denmark). The plates were placed inside a plate reader (FLUOstar Omega, BMG Labtech, Offenburg, Germany) and incubated for 18 h at 37°C, and kinetic measurements were taken every 30 min at OD_600__nm_. The assays (dose 5 vs. Ctrl, and dose 60 vs. Ctrl) were carried out with sample duplicates and in three independent experiments (*n* = 3).

The mean maximum growth rate (μ_*max*_) and generation time were calculated from each replicate growth curve (six replicate growth curves per sample type) using at least five consecutive time points. The mean and standard error of the mean for each sample (*n* = 3) were calculated.

##### Viability of planktonic population: live/dead fluorescence

After 18 h of culture, the plate was removed from the plate reader; 100 μL of the supernatant was transferred to a Costar black microtiter 96-well plate (Corning Incorporated, Kennebunk, ME, United States) and stained with 100 μL per well of a solution containing SYTO9 and propidium iodide from a LIVE/DEAD Baclight Viability kit (Invitrogen, Life Technologies, Carlsbad, CA, United States) following the manufacturer’s instructions. After 15 min of incubation, the fluorescence intensity was measured in a plate reader. The excitation/emission for green and red channels were 485/530 nm and 485/630 nm, respectively.

##### Adhesion to polystyrene: microtiter plate assay

After the removal of the supernatant, the plate was inverted and rinsed by submersion in saline to remove non-adherent bacteria. The remaining adhered bacterial cells were stained with crystal violet according to a previously described protocol (Zaborowska et al., 2017). In brief, cells were stained with 0.1% crystal violet solution and incubated for 10 min. Thereafter, the plate was rinsed and eluted in 95% ethanol for 15 min. The solution was transferred to a new 96-well plate, and the absorbance was measured at 595 nm in a plate reader.

### Addition of EVs From a Biofilm^*pos*^/GEN^*R*^ Strain (EV Donor) to a Biofilm^*neg*^/GEN^*S*^ Strain (Recipient Cell)

#### Effect on Antimicrobial Tolerance and Adhesion to Polystyrene

In another subset of experiments (Figure 1B), EVs derived from the biofilm^*pos*^/GEN^*R*^ strain *S. epidermidis* ATCC 35984 were isolated as described above, with the exception that the strain was cultured in tryptic soy broth (TSB; Eur. Pharm. Scharlau, Spain) without gentamicin.

Isolated colonies from an overnight blood agar culture of *S. epidermidis* ATCC 64523 were suspended in 4 mL of MHB until an OD_546__nm_ of 0.25, equivalent to 10^8^ CFU × mL^–1^, was reached. A total volume of 200 μL per well, consisting of 100 μL of the bacterial suspension (10^5^ CFU × mL^–1^ final concentration per well), 50 μL of isolated EVs from *S. epidermidis* ATCC35984 (final well concentrations of 0 or 100 μg × mL^–1^), and 50 μL of three different concentrations of GEN (0, 0.03, 0.12, and 0.5 μg × mL^–1^ final well concentrations) were dispensed into Nunc 96-well plates. In addition, 5 × 10^5^ CFU × mL^–1^ of the *S. aureus* ATCC 29213 strain was used as a quality control for the GEN concentrations, and the *S. epidermidis* ATCC 35984 and ATCC 12228 strains were used for the microtiter plate assay as positive and negative Ctrls, respectively. The plate was placed in a plate reader and incubated for 18 h at 37°C, and kinetic measurements were recorded every 30 min at OD_600__nm_. The assay was carried out with sample triplicates, and three independent experiments were performed (*n* = 3). Live/dead staining of planktonic bacteria and the microtiter plate assay on adhered bacteria was performed after 18 h of culture, as described above.

#### Effect on Adhesion to Glass

The inoculum of *S. epidermidis* ATCC 64523 suspended in MHB was prepared as described above, and a final concentration of 5 × 10^5^ CFU × mL^–1^ was added to each well of an 8-well glass slide (Millicell EZ SLIDE, Millipore, Darmstadt, Germany). The bacterial suspensions were supplemented with and without 100 μg × mL^–1^ EVs derived from the biofilm^*pos*^/GEN^*R*^ *S. epidermidis* ATCC 35984 strain and incubated for 5 h and 24 h. Non-adherent bacterial cells were removed by rinsing three times with saline. The samples were stained with a Filmtracer LIVE/DEAD Biofilm Viability kit (Invitrogen, Life Technologies, Carlsbad, CA, United States) for 30 min and rinsed before adding mounting media and cover slides. The samples were visualized with a Nikon C2 confocal laser-scanning microscope (CLSM, Nikon, Tokyo, Japan). The experiment was performed three times (*n* = 3).

### Statistics

One-way ANOVA followed by Dunnett’s *post hoc* test was performed to evaluate significant differences between the Ctrl and the EVs isolated under different gentamicin concentrations for the area under the growth curve (AUC), μ_*max*_, generation time, adhered biomass, and live/dead fluorescence readings. One-way ANOVA followed by an LSD (least significant difference) *post hoc* test was performed to analyze differences in the EV characteristics, AUC, μ_*max*_, generation time, adhered biomass, and live/dead fluorescence readings of the different EV groups (EV_1_, EV_2_, and EV_3_). A *t*-test was performed to calculate differences between Ctrl and EVs from the biofilm^*pos*^/GEN^*R*^ strain. Statistical analyses were performed using SPSS Statistics 21 (IBM Corporation, United States), and the significance level was set at *p* < 0.05.

## Results

### Vesiculation Under Subinhibitory Concentrations of GEN and the Function of These EVs in Bacterial Survival and Adhesion Under Antimicrobial Selective Pressure

#### EV Isolation and Characterization

**FIGURE 2:**
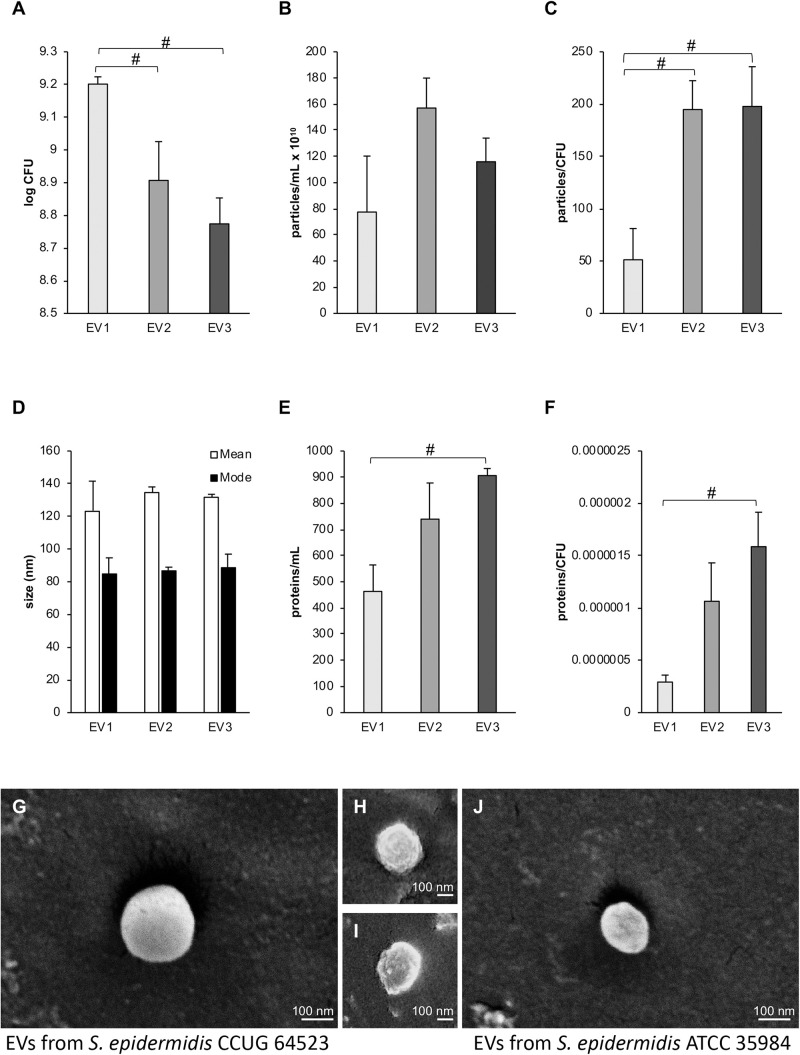
Influence of subinhibitory culture conditions on vesiculation. **(A)** CFUs per mL of *Staphylococcus epidermidis* CCUG 64523 cultures supplemented with different gentamicin concentrations used for EV isolation. **(B)** Particles per mL for the different EV isolation conditions as determined by NTA. **(C)** Particles per CFU. **(D)** The mean and mode sizes of particles as determined by NTA. **(E)** The protein levels for each EV type in μg × mL^–1^, demonstrating significantly higher protein levels in EV_3_ than in EV_1_ (*p* < 0.05). **(F)** Protein (μg × mL^–1^) per CFU, and significantly higher amounts of proteins per CFU were observed in EV_3_ than in EV_1_ (*p* < 0.05). EV_1_, EV_2_, and EV_3_, are EVs isolated in the presence of 0, 0.03, and 0.06 μg × mL^–1^ gentamycin, respectively. EVs were isolated in triplicate for each EV type, and the bars represent the mean ± SEM; ^#^ indicates a significant difference between different groups of EVs. **(G–I)** Representative scanning electron micrographs of EVs from *S. epidermidis* CCUG 64523 isolated in the presence of **(G)** 0, **(H)** 0.03, and **(I)** 0.06 μg × mL^–1^ gentamycin. **(J)** EVs isolated from *S. epidermidis* ATCC 35984 cultured in tryptic soy broth.

Viability counting performed with the three *S. epidermidis* cultures supplemented with increasing concentrations of GEN (0, 0.03, and 0.06 μg × mL^–1^) showed a significant decrease in CFU (CFU_0__GEN_ > CFU_0.__03__*GEN*_; CFU_0__GEN_ > CFU_0.__06__*GEN*_) (Figure 2A). The mean concentration of the isolated EVs (particles per mL) measured by NTA was 7.75 × 10^10^ for EV_1_, 1.57 × 10^11^ for EV_2_, and 1.16 × 10^11^ for EV_3_ (Figure 2B). A significant increase in the number of EVs per CFU was observed for EV_2_ and EV_3_ in comparison to EV_1_ (Figure 2C). An NTA was employed to determine the mean and mode sizes of the isolated EVs. The addition of GEN did not have an effect on the mean sizes of the isolated EVs (mean 123.5 ± 18 nm, 134.7 ± 3 nm, and 131.8 ± 22 nm and mode 84.5 ± 20 nm, 87.0 ± 2 nm, and 88.8 ± 8 nm for EV_1_, EV_2_, and EV_3_, respectively; Figure 2D).

The total amount of protein contained in EV_1_, EV_2_, and EV_3_ was 463.3 ± 101.3 μg × mL^–1^, 739.7 ± 138.1 μg × mL^–1^, and 906.3 ± 26.3 μg × mL^–1^, respectively. The total protein concentration and the EV protein concentration per CFU were significantly higher in vesicles isolated from strains cultured with subinhibitory concentrations of 0.06 μg × mL^–1^ GEN (EV_3_) than in vesicles isolated from Ctrl cultures without GEN (EV_1_) (*p* = 0.02 and 0.018, respectively) (Figures 2E,F).

Extracellular vesicles from the three GEN culture conditions were detected and visualized by SEM (Figures 2G–I). Overall, EVs appeared spherical with some external irregularities due to sample preparation and the electron beam effect.

#### Function of EVs Isolated in the Presence of Subinhibitory Gentamicin Concentrations in Growth, Survival, and Adhesion

**FIGURE 3:**
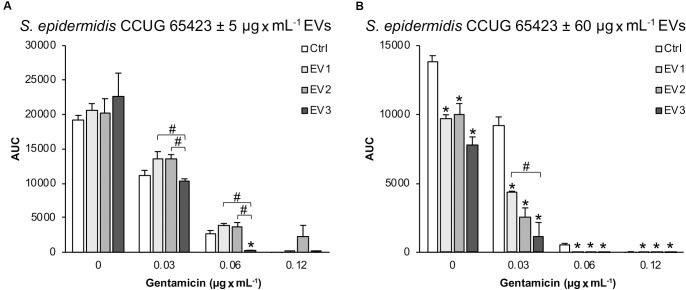
The total growth of *Staphylococcus epidermidis* CCUG 64523 in four different culture conditions (0, 0.03, 0.06, and, 0.12 μg × mL^–1^ GEN) calculated as the AUC after 18 h of culture with **(A)** 5 μg × mL^–1^ EVs and **(B)** 60 μg × mL^–1^ EVs. The data represent the mean ± SEM (*n* = 3); *indicates a significant difference compared to the Ctrl; ^#^ indicates a significant difference between different EV-stimulated groups with *p* < 0.05.

Under the 0 GEN culture conditions, the total bacterial growth of the clinical strain, calculated as the AUC, after stimulation with 5 μg × mL^–1^ EVs of all types (EV_1_, EV_2_, and EV_3_) was similar to that of the Ctrl (Figure 3A). However, in the presence of 0.03 μg × mL^–1^ GEN, compared with the EV_1_ and EV_2_ treatments, the EV_3_ treatment significantly decreased the total growth, and in the presence of 0.06 μg × mL^–1^ GEN, compared with the Ctrl, EV_1_ and EV_2_ treatments, the EV_3_ treatment decreased the total growth (Figure 3A). In the presence of 0.12 μg × mL^–1^ GEN (MIC), bacterial growth was equally inhibited for all groups except for the EV_2_ group, which showed increased cell survival at the MIC GEN dose.

In contrast, stimulation with 60 μg × mL^–1^ EVs of all three types significantly decreased the total growth of the clinical strain compared with the unstimulated Ctrl strain in all culture conditions (Figure 3B). In addition, a significant difference in *S. epidermidis* growth was observed between the EV_1_- and EV_3_-stimulated groups in the presence of 0.03 μg × mL^–1^ GEN.

**FIGURE 4:**
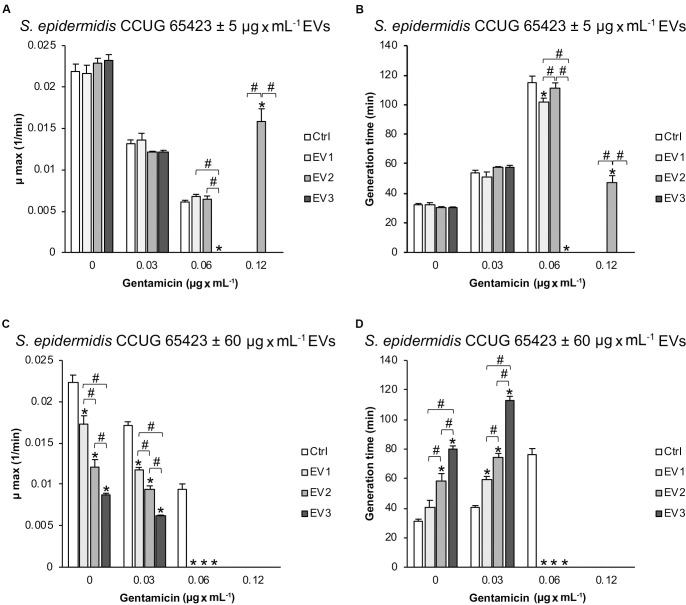
Calculated **(A–C)** maximum growth rate (μmax; min^–1^) and **(B–D)** generation time (min) for the different growth curves of *Staphylococcus epidermidis* CCUG 64523 stimulated with **(A,B)** 5 μg × mL^–1^ EVs and **(C,D)** 60 μg × mL^–1^ EVs. The data represent the mean ± SEM (*n* = 3); * indicates a significant difference compared to the Ctrl; ^#^ indicates a significant difference between different EV-stimulated groups with *p* < 0.05.

The maximum growth rate (μ_*max*_, min^–1^) and generation time (min) for the growth curves obtained for the different groups are shown in Figure 4. The μ_*max*_ and generation times were not affected by stimulation with 5 μg × mL^–1^ EVs in the presence of 0 and 0.03 μg × mL^–1^ GEN (Figures 4A,B). However, compared to that in all groups, the growth rate in the EV_3_ group (0.06 μg × mL^–1^ GEN) was significantly lower, but in the EV_2_ group (0.12 μg × mL^–1^ GEN), the growth rate was significantly higher. The generation time for the EV_1_ treatment group was also reduced compared with the Ctrl treatment group in the presence of 0.06 μg × mL^–1^ GEN and for the EV_2_ group compared with all groups at 0.12 μg × mL^–1^ GEN (Figure 4B). The μ_*max*_ and generation time of EV_3_ at 0.06 μg × mL^–1^ GEN and of Ctrl, EV_1_, and EV_3_ at 0.12 μg × mL^–1^ GEN were 0. In addition, there were significant differences between the generation times of cells treated with EVs of different origins (EV_3_ < EV_1_; EV_3_ < EV_2_; EV_1_ < EV_2_) (Figure 4B).

Compared with that of the unstimulated Ctrl *S. epidermidis*, the μ_*max*_ of *S. epidermidis* stimulated with the three EV types at 60 μg × mL^–1^ decreased (and generation time increased) significantly in a dose-dependent manner at 0 μg × mL^–1^ GEN and 0.03 μg × mL^–1^ GEN (Figures 4C,D). The EV groups did not grow at GEN concentrations of 0.06 and 0.12 μg × mL^–1^, whereas the Ctrl group did grow in the presence of 0.06 μg × mL^–1^ GEN (Figures 3B, 4C,D).

The average mean growth curves for the different culture conditions are provided in Supplementary Material 1, 2.

**FIGURE 5:**
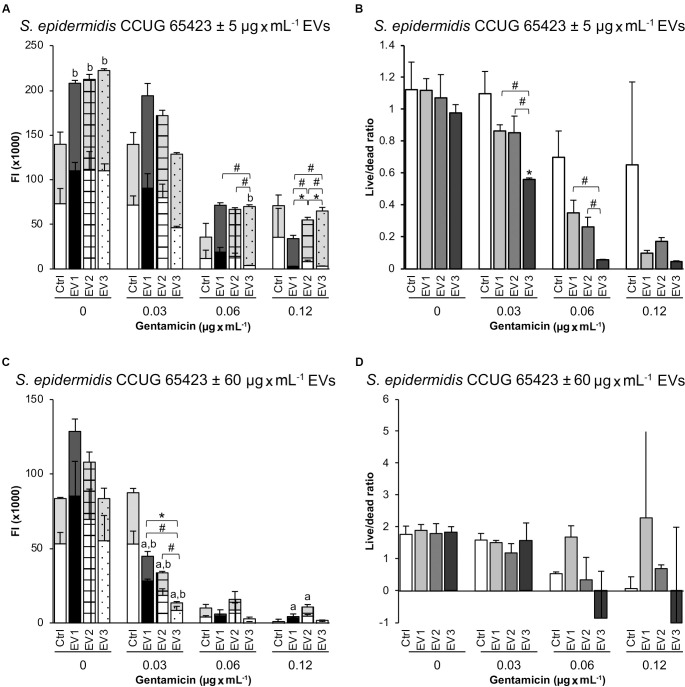
Live and dead populations of planktonic *Staphylococcus epidermidis* after 18 h measured as fluorescence intensity (FI) in a plate reader. **(A,B)** Treatment with EVs (5 μg × mL^–1^). **(C,D)** Treatment with EVs (60 μg × mL^–1^). The data represent the mean ± SEM (*n* = 3) of live and dead cells. Gray background bars (top) represent the dead population, and white background bars (bottom) represent the live population. Asterisks and hashtags indicate statistically significant differences between live and dead populations, respectively. Letters a (live population) and b (dead population) indicate a significant difference from the untreated Ctrl (*p* < 0.05).

The viability of the planktonic phase measured by live/dead fluorescence staining showed no differences between the Ctrl and EV-treated groups (5 μg × mL^–1^) at any of the GEN concentrations (Figure 5A). However, there was a significant increase in dead bacteria in all EV-treated groups compared with the Ctrl group in the presence of 0 μg × mL^–1^ GEN and in the EV_3_ group compared with the Ctrl group in the presence of 0.06 μg × mL^–1^ GEN (Figure 5A). An increase in the dead population in the presence of 0.06 μg × mL^–1^ GEN was observed between the EV_1_- and EV_3_-treated groups (dead: EV_3_ > EV_1_) and between the EV_2_- and EV_3_-treated groups (dead: EV_3_ > EV_2_). A significant increase in the dead population was detected for all the EV-treated groups at 0.12 μg × mL^–1^ GEN (dead: EV_1_ < EV_2_ < EV_3_). A significant difference in the live population of the EV-treated groups was observed at 0.12 μg × mL^–1^ GEN (live: EV_1_ < EV_2_, and EV_2_ > EV_3_) (Figure 5A). The live/dead ratio was significantly decreased in the EV_2_ group compared with the Ctrl group, in the EV_3_ group compared with the EV_1_ and EV_2_ groups in the presence of 0.03 μg × mL^–1^ GEN and in the EV_3_ group compared with the EV_1_ and EV_2_ groups in the presence of 0.06 μg × mL^–1^ GEN (Figure 5B). The addition of 60 μg × mL^–1^ EVs induced a significant decrease in both live and dead populations for all EV-treated groups compared with the Ctrl group at 0.03 μg × mL^–1^ GEN and in the live population (live: Ctrl < EV_1_, Ctrl < EV_2_) at 0.12 μg × mL^–1^ GEN (Figures 5C,D). Differences between the EV-treated groups were observed for the live population (live: EV_1_ > EV_3_) and dead population (dead: EV_1_ > EV_3_, EV_2_ > EV_3_) at 0.03 μg × mL^–1^ GEN (Figure 5C). No significant differences were detected in the live/dead ratio when cells were stimulated with 60 μg × mL^–1^ EVs (Figure 5D).

**FIGURE 6:**
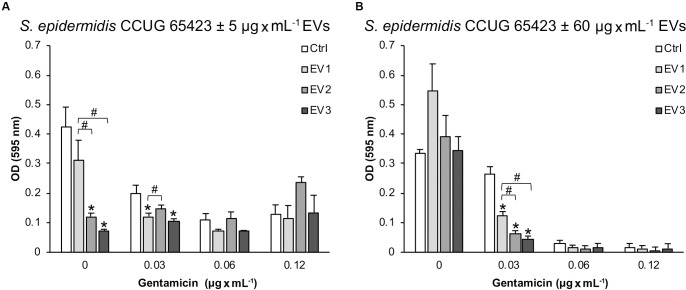
The adherence of *Staphylococcus epidermidis* CCUG 64523 cells to tissue culture plates as determined by microtiter plate assay after 18 h of incubation with **(A)** 5 μg × mL^–1^ EVs and **(B)** 60 μg × mL^–1^ EVs. The data represent the mean ± SEM (*n* = 3); * significant difference compared to the Ctrl; ^#^ significant difference between different groups of EVs.

A microtiter plate assay was performed to investigate the role of EVs isolated from *S. epidermidis* CCUG 64523 under GEN selective pressure in cell adhesion of the same strain cultured with and without GEN to polystyrene. Overall, compared with that of the untreated Ctrl cells, the adhesion of the cells treated with 5 μg × mL^–1^ EVs was reduced by more than 20%. Compared with that of the unstimulated Ctrl cells, the adhesion of the cells treated with EV_2_ and EV_3_ was significantly reduced by 71% and 82%, respectively, at 0 μg × mL^–1^ GEN (Figure 6A). A significant decrease in adhesion was also observed in the EV_3_ group compared with the EV_1_ group and in the EV_2_ group compared with the EV_1_ group. The adhesion was reduced significantly in the EV_1_- and EV_3_-stimulated groups (39% and 47% reduction, respectively) compared with the Ctrl group, and the adhesion of the EV_3_-treated group was reduced compared to that of the EV_2_-treated group at 0.03 μg × mL^–1^ GEN (Figure 6A). Equivalent adhered biomass was quantified for all groups in the presence of 0.06 and 0.12 μg × mL^–1^ GEN (Figure 6A).

Culturing of *S. epidermidis* without GEN resulted in similar adhered biomass levels for all groups (60 μg × mL^–1^ EVs) (Figure 6B). However, with the addition of 0.03 μg × mL^–1^ GEN, compared with the Ctrl group, the adhesion to polystyrene of all EV-stimulated groups (60 μg × mL^–1^) was significantly reduced (54%, 77%, and 83% for EV_1_, EV_2_, and EV_3_, respectively) (Figure 6B). In addition, the EV_2_- and EV_3_-stimulated groups adhered significantly less well to polystyrene than the EV_1_ group (Figure 6B). No differences in adhesion were detected in the presence of 0.06 and 0.12 μg × mL^–1^ GEN (Figure 6B).

#### Effect of EVs From a Biofilm^*pos*^/GEN^*R*^ Strain (EV Donor) on the Antimicrobial Tolerance of a Biofilm^*neg*^/GEN^*S*^ Strain (Recipient Cells)

The EVs isolated from the biofilm^*pos*^/GEN^*R*^ strain *S. epidermidis* ATCC 35984 used for the antimicrobial tolerance experiments had a protein concentration of 5500 μg × mL^–1^ as measured by Nanodrop and showed a typical round morphology when visualized by SEM (Figure 2J).

**FIGURE 7:**
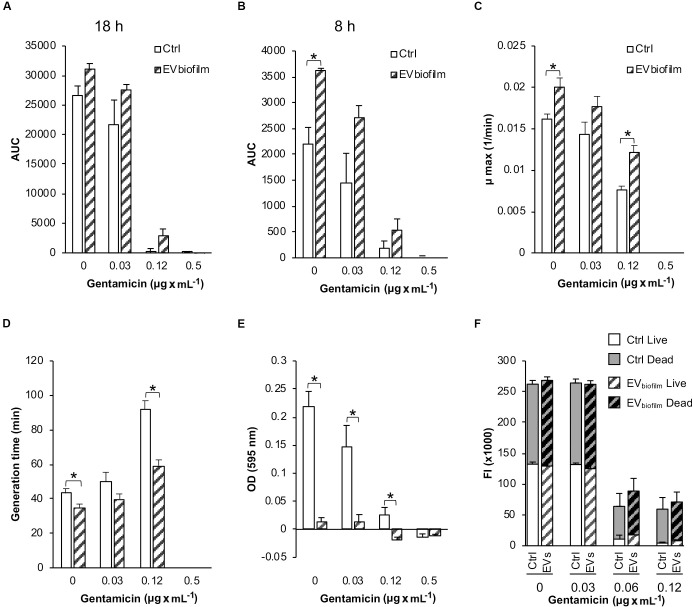
Bacterial growth, cell adhesion, and viability of the biofilm^*neg*^/GEN^*S*^ *Staphylococcus epidermidis* CCUG 64523 upon stimulation with EVs derived from the biofilm^*pos*^/GEN^*R*^ strain *S. epidermidis* ATCC 35984 (EV_*biofilm*_). The total growth was calculated as the AUC after **(A)** 18 h of culture and **(B)** 8 h of culture. **(C)** The maximum growth rates. **(D)** Generation times. **(E)** Cell adhesion as measured by the microtiter plate assay. **(F)** Live/dead fluorescence measurements of viability of the planktonic population. Gray background bars (top) represent the dead population, and white background bars (bottom) represent the live population. The data represent the mean ± SEM (*n* = 3); * indicates a significant difference compared to the Ctrl with *p* < 0.05.

Overall, the addition of EVs derived from a biofilm^*pos*^/GEN^*R*^ *S. epidermidis* strain (EV_*biofilm*_) to a recipient biofilm^*neg*^/GEN^*S*^ *S. epidermidis* strain increased the growth of the recipient cells relative to that of the untreated Ctrl cells under all the different culture conditions, although the difference was significant only after 8 h for the cultures containing 0 and 0.12 μg × mL^–1^ GEN (Figures 7A,B). Compared with the untreated Ctrl, the EV_*biofilm*_ treatment significantly increased the maximum growth rate and consequently decreased the generation time (8–33 min shorter) in the presence of 0 and 0.12 μg × mL^–1^ GEN (Figures 7C,D).

Mean growth curves for this experiment are provided in Supplementary Material 3.

Next, we investigated the effect of EV_*biofilm*_ treatment on the adhesion of a biofilm^*neg*^/GEN^*S*^ clinical *S. epidermidis* strain to polystyrene. Compared with the untreated Ctrl, EV_*biofilm*_ treatment significantly inhibited bacterial adhesion to the tissue culture plate under all of the different culture conditions, except in the presence of 0.5 μg × mL^–1^ GEN where growth was inhibited (Figure 7E). Compared with the untreated Ctrl, EV_*biofilm*_ treatment reduced the biofilm biomass up to 90% (for all culture conditions below the MIC). The viability of the planktonic phase measured by live/dead fluorescence staining did not reveal any differences between the Ctrl and EV-treated groups for any of the culture conditions (Figure 7F).

#### Effect of EVs From a Biofilm^*pos*^/GEN^*R*^ Strain on the Adhesion of a Biofilm^*neg*^/GEN^*S*^ Strain to Glass

**FIGURE 8:**
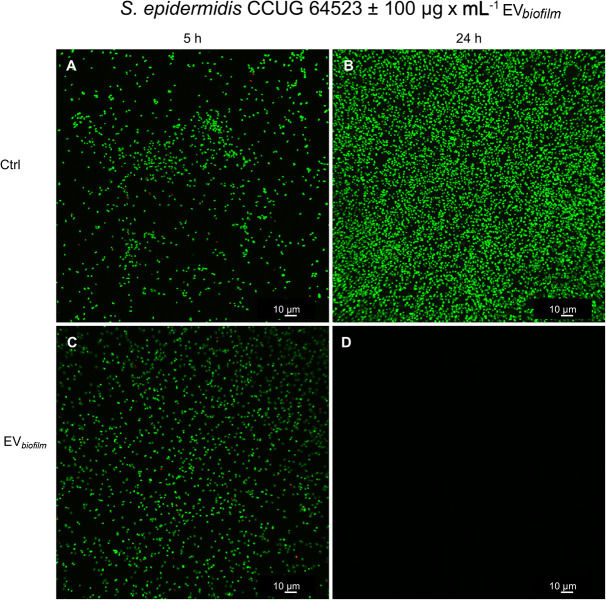
Confocal laser-scanning micrographs showing the adhesion of *S. epidermidis* CCUG 64523 cells to glass slides. **(A,B)** Unstimulated Ctrl cells cultured for **(A)** 5 h and **(B)** 24 h and **(C,D)** cultures supplemented with EVs (100 μg × mL^–1^) derived from the biofilm-producing/GEN^*R*^ strain *S. epidermidis* ATCC 35984 (EV_*biofilm*_) and cultured for **(C)** 5 h and **(D)** 24 h. Bacterial cells were stained with SYTO9; live cells were stained green, and propidium iodide was used to stain dead cells red. All images were taken using both channels. Mainly live (green) cells were observed, and only a few scattered dead (red) cells were found. Scale bar: 10 μm.

Representative confocal images of the *in situ* adherence of the *S. epidermidis* CCUG 64523 strain to glass slides after 5 h and 24 h are shown in Figure 8. The unstimulated Ctrl after 5 h and 24 h is shown in Figures 8A,B, respectively. The adhesion of *S. epidermidis* cells upon stimulation with EV_*biofilm*_ for 5 h and 24 h is shown in Figures 8C,D, respectively. No difference was observed between the Ctrl and EV-stimulated groups at 5 h; however, a drastic decrease in the quantity of cells adhered to glass was observed in the EV-stimulated group after 24 h, and practically empty surfaces were observed. For both groups and timepoints, the majority of cells were alive (green), and only a few single dead cells (red) were observed.

## Discussion

*Staphylococcus epidermidis* commonly causes implant-associated infections, and its main virulence mechanism involves forming biofilms, which adhere to implant surfaces. Adhered bacteria are difficult to eradicate, and long-term antimicrobial treatment is often necessary. However, long-term antimicrobial treatment may be suboptimal, making the pathogen resistant or tolerant to treatment. In this study, we evaluated whether the antimicrobial pressure exerted by subinhibitory concentrations of gentamicin could affect vesiculation as a mechanism for antimicrobial tolerance and survival and affect cell attachment to surfaces. Culturing in the presence of subinhibitory concentrations of GEN resulted in an increased number of EVs of similar size that contained a higher concentration of proteins compared to the EVs from Ctrl culture conditions. These findings suggest that vesiculation is a survival response to subinhibitory concentrations of gentamicin.

We could not completely rule out the possibility that compounds other than EVs originating from lysed cells were obtained during EV isolation. However, the increase in EV-associated protein levels observed in cultures with subinhibitory concentrations of GEN is unlikely to result from cell lysis since the number of isolated EVs was reduced when they were isolated from cultures containing the MIC dose of 0.12 μg × mL^–1^ GEN (data not shown). Schooling et al. (2009) showed that OMVs bind to gentamicin and proposed that OMVs could act as “sponges or decoys” to reduce antimicrobial agents before they affect cells (Schooling and Beveridge, 2006). In the present study, this scenario could be a possibility because of the observed survival effect against GEN exerted by EV_*biofilm.*_ However, the result showed that cells treated with 60 μg × mL^–1^ EVs showed less total growth than those cultured with 5 μg × mL^–1^ EVs in the presence of GEN is contrary to this mechanism of action. Furthermore, gentamicin and other aminoglycoside antibiotics are potent inhibitors of protein synthesis in a wide range of bacteria (Kadurugamuwa et al., 1993), and it could be hypothesized that under the selective pressure of GEN, *S. epidermidis* cells react by packaging increased quantities of proteins inside EVs, especially those that aid survival under adverse conditions.

Very little information is available in the literature on gram-positive vesiculation, and much of the knowledge is extrapolated from gram-negative OMVs. Hypervesiculation may be an induced innate immune bacterial defense mechanism against antimicrobial agents targeting the outer membrane (Manning and Kuehn, 2011). The overproduction of OMVs by gram-negative bacteria increases the resistance of cells to different antimicrobial agents and increases the virulence of the cells since OMVs can transfer and deliver virulence factors to recipient human cells (Beceiro et al., 2013). Studies on *Pseudomonas aeruginosa* vesiculation under antimicrobial pressure and aquatic bacteria under UV stress (Gamalier et al., 2017) have also found increased vesiculation in the presence of antimicrobials; the increased OMV protein levels most likely reflect vesiculation stimulation and are not due to cell lysis (Kadurugamuwa and Beveridge, 1997; Maredia et al., 2012). Kadurugamuwa and coworkers concluded that increased vesiculation under antimicrobial pressure might have important clinical implications in the treatment of patients with cystic fibrosis infected with β-lactam-resistant *P. aeruginosa* strains since proteomic analyses showed the presence of β-lactam in OMVs (Ciofu et al., 2000). McBroom and Kuehn (2007) showed that increased vesiculation correlates positively with the survival of gram-negative bacteria upon exposure to chemical stress.

In addition, there is evidence that OMVs are involved in the aggregation of bacterial cells (*Bacteroides gingivalis* vesicles bind and aggregate *Actinomyces viscosus* cells) (Ellen et al., 1989) and in biofilm development (Schooling and Beveridge, 2006; Baumgarten et al., 2012). Recent studies demonstrated that subinhibitory doses of antimicrobials can enhance *P. aeruginosa* biofilm formation (Bagge et al., 2004; Lucas et al., 2005). These results suggest that increased biofilm formation in the presence of antimicrobials may be a defense mechanism of the bacteria against antibiotic stress. In the present study, the opposite outcome was observed: in the presence of gentamicin, EVs from two different *S. epidermidis* strains affected the adhesion of cells to different degrees by mainly decreasing the amount of total adhered biomass after 18 h of culture. Considering this finding and the fact that growth conditions can have a drastic effect on OMV production and composition (Bauman and Kuehn, 2006; Kulp and Kuehn, 2010), OMVs and EVs might play an important role in the development of biofilms and in their increased antimicrobial resistance (Schooling and Beveridge, 2006). Another study revealed that drug-binding proteins were more concentrated in biofilm-derived OMVs than planktonic-derived OMVs, indicating the involvement of OMVs in biofilm development and antimicrobial resistance (Ciofu et al., 2000). It has been found that the addition of exogenous DNA to *P. aeruginosa* biofilms results in a 2-fold increased level of resistance to gentamicin, and it is also known that eDNA may be derived endogenously from OMVs among other sources (Chiang et al., 2013).

The number of EVs released by *S. epidermidis* and their total protein content increased during exposure to subinhibitory concentrations of gentamicin. These EVs had various concentration-dependent effects on cell growth. With EVs at a dose of 5 μg × mL^–1^, the total growth of the clinical strain after 18 h was similar to that of the untreated Ctrl. However, compared with that of the Ctrl, the total growth of cells treated with 60 μg × mL^–1^ EVs was dramatically decreased in all culture conditions. While the total growth of the Ctrl group (no stimulation) remained constant in both experiments with the two EV doses, the total growth of the groups stimulated with EVs at a dose of 60 μg × mL^–1^ was approximately half that of the group stimulated with EVs at a dose of 5 μg × mL^–1^. This decrease in total growth after the addition of 60 μg × mL^–1^ EVs is explained by the significant decrease in the maximum growth rate and is not due to changes in viability, as determined by the fluorescence readings (generally, the proportion of live cells was higher than that of dead cells). Decreasing the maximum growth rate has been suggested as a mechanism of antimicrobial tolerance in bacteria, especially in biofilms, and could explain the effect of 60 μg × mL^–1^ EVs on cells as a mechanism of gentamicin survival (Hall and Mah, 2017). This potential survival mechanism of decreasing the growth rate was further exemplified when EVs at a dose of 60 μg × mL^–1^ were added to cells cultured with the MIC of 0.12 μg × mL^–1^ GEN, where the EV-treated groups (EV_1_ and EV_2_) did not display any growth and showed a larger live cell population than the Ctrl group. Therefore, EVs isolated from the GEN^*S*^/biofilm^*neg*^ clinical *S. epidermidis* strain enabled the cells of the same strain to survive at the MIC by neither growing nor dying in the presence of GEN but by decreasing the maximum growth rate, demonstrating tolerance to the antimicrobial agent.

When EVs at a dose of 5 μg × mL^–1^ were added to cultures containing 0.06 μg × mL^–1^ GEN, all groups except the EV_3_ group experienced growth and had similar viability as the Ctrl group. However, the EV_3_ group did not show growth and displayed the largest amount of dead cells, which could indicate the presence of different cargos in the EVs isolated from the cells in the presence of 0.06 μg × mL^–1^ GEN (EV_3_). Another interesting finding is that at the MIC of 0.12 μg × mL^–1^ GEN, only the *S. epidermidis* cells in the EV_2_ group (5 μg × mL^–1^ dose) showed growth, as indicated by the increase in μ_*max*_. The adhesion of *S. epidermidis* cells to the tissue culture plate decreased only in the presence of 0 and 0.03 μg × mL^–1^ GEN upon stimulation with 5 and 60 μg × mL^–1^ EVs.

With exceptions, all these results indicate that EVs derived from the clinical biofilm^*neg*^/GEN^*S*^ *S. epidermidis* strain contributed to similar (5 μg × mL^–1^ EV dose) or decreased (60 μg × mL^–1^ EV dose) overall growth due to the decreased maximum growth rate, slower planktonic cell division, and, in some instances, reduced adhesion events. These results also indicate that depending on the concentration of EVs and consequently their protein concentration, the mechanisms for surviving subinhibitory concentrations of antibiotics could also be different. These effects could also be species- and strain-dependent since the supplementation of *S. aureus* cultures in media other than MHB with *S. aureus*-derived EVs did not significantly influence growth (Askarian et al., 2018). A recent study demonstrated similar results to ours and showed that the addition of EVs from *S. aureus* resulted in reduced adhesion to the tissue culture plate, possibly because the surface became coated by EVs, making it more hydrophilic and less reachable by the bacteria (Im et al., 2017). This could, at least partly, be an explanation for the radical decrease in *S. epidermidis* adhesion to glass that was observed by confocal microscopy after 24 h in the presence of EVs (EV_*biofilm*_).

The present study also addressed whether vesicles from an antimicrobial-resistant and biofilm-producing strain could change the phenotype of a non-biofilm-producing and susceptible strain. Compared with the unstimulated Ctrl, the *S. epidermidis* cells treated with EVs from a biofilm^*pos*^/GEN^*R*^ strain demonstrated a significant increase in growth and cell division events during the exponential phase, which is explained by an increased maximum growth rate and faster doubling time (8–33 min shorter), as well as drastically reduced cell adhesion after 18 h. This growth-promoting effect has been observed previously by our group in *S. epidermidis* ATCC 35984 and *S. aureus* cultured without gentamicin (unpublished results). Whole genome sequencing and proteomic analyses of donor EVs isolated from *S. epidermidis* ATCC 35984, performed by our group, showed the presence of the gentamycin resistance gene *aac(6*′*)-aph(2*′′) and the 6′-aminoglycoside N-acetyltransferase/2′′-aminoglycoside phosphotransferase, which is the protein responsible for this resistance (Shaw et al., 1993) (unpublished results). However, we did not evaluate whether the *aac(6*′*)-aph(2*′′) gene or the 6′-aminoglycoside N-acetyltransferase/2′′-aminoglycoside phosphotransferase from the donor EVs was transferred, and ongoing studies are being pursued in this direction. The aminoglycoside N-acetyltransferase carried by the EVs confers gentamicin resistance by enzymatic inactivation of the antibiotic molecule and by sequestration of the drug via tight binding, explaining the increase in growth observed in the EV_*biofilm*_ group in this study.

Outer membrane vesicles have been shown to disseminate virulence and resistance factors and to mediate horizontal transfer of plasmids harboring antibiotic resistance genes (Rumbo et al., 2011). EVs from *S. aureus* containing β-lactamase enable other ampicillin-susceptible gram-negative and gram-positive bacteria to survive in the presence of ampicillin (Lee et al., 2013). In the latter study, the *blaZ* gene conferring penicillin resistance was not detected in the EVs.

Although the EVs from the biofilm^*pos*^/GEN^*R*^ strain did not transform the recipient susceptible strain into a GEN^*R*^ strain (no phenotypic shift in the GEN MIC value), the EVs contributed to increased tolerance of the susceptible strain to GEN by inducing an overall growth increase effect. Unlike other transformation experiments where the cell suspension mixed with purified vesicles was incubated overnight (Yaron et al., 2000; Rumbo et al., 2011), in our experiment, different concentrations of gentamicin were added directly after the addition of the extracted vesicle solution; presumably, the timing of this consecutive addition could have been insufficient for the *S. epidermidis* cells to incorporate the EVs. Therefore, an increased exposure time between the bacterial cells and the EVs might elicit different effects than those observed in this study. Moreover, different culture conditions could also affect the assimilation of the vesicles.

Previous studies indicated that EVs from fungi can synthesize melanin, a polymer contained in their cell wall, *in situ*, proposing that EVs could be retained in the bacterial wall to provide compounds necessary for cell wall synthesis and maintenance (Brown et al., 2015). Consistent with this hypothesis, the observed growth promotion effect resulting from the addition of *S. epidermidis* EV_*biofilm*_ could be partially explained by the same mechanism, whereby the EVs facilitate cell growth and decrease the time or energy required for division. Further studies should be performed to confirm this hypothesis (Wikler et al., 2007). In another study, OMVs from gram-negative bacteria were reported to fuse with both gram-negative and gram-positive bacteria (Li et al., 1998). This ability to integrate into membranes could have a similar effect of increasing cell division by forming structures from the outer membrane and lumen in the recipient bacteria.

Clinical studies have shown that *S. aureus* and *S. epidermidis* are the most common species isolated from infected percutaneous bone-anchored amputation prostheses and hearing aids (Lenneras et al., 2017; Trobos et al., 2018). *Staphylococcus* spp. isolated from these patients have the ability to secrete EVs containing DNA, RNA and proteins *in vitro* and have a cytotoxic effect on host cells (unpublished results). The increase in the number of EVs released by *S. epidermidis* during exposure to gentamicin might have important clinical implications for the treatment of patients with implant-associated infections caused by *S. epidermidis*.

## Conclusion

The results from this study showed that subinhibitory concentrations of gentamicin increase the number and total protein content of secreted EVs. In addition, these EVs regulate the growth of *S. epidermidis* cells as well as influence cell attachment to surfaces, affecting survival under antimicrobial pressure conditions. Increased vesiculation during exposure to antimicrobial agents could have clinical implications for the treatment of patients with implant-associated infections caused by *S. epidermidis*.

Extracellular vesicles from *S. epidermidis* decrease cell adhesion to polystyrene and glass, and, depending on their strain of origin and concentration, EVs may function as survival factors by promoting growth via the shortening of the lag phase and generation time. Consequently, *S. epidermidis* cells may be forced to prioritize cell division over other functions (i.e., cell adhesion). The molecular mechanisms behind these effects are still undetermined, and the next step will be to identify plausible mechanisms.

## Data Availability Statement

All datasets generated for this study are included in the article/Supplementary Material.

## Author Contributions

MT was involved in the design of the study. MZ, CT, FV, and FS were involved in the execution of the study. MZ, CT, and MT were involved in the analysis. MZ, PT, and MT were involved in the interpretation of the results. MZ and MT wrote the primary manuscript. All authors reviewed and edited the manuscript.

## Conflict of Interest

The authors declare that the research was conducted in the absence of any commercial or financial relationships that could be construed as a potential conflict of interest.
