# Supplementary material for: Extracellular Vesicles Influence the Growth and Adhesion of Staphylococcus epidermidis Under Antimicrobial Selective Pressure
Source: Front Microbiol. 2020 Jul 2;11:1132. doi: 10.3389/fmicb.2020.01132 (PMC7346684; doi:10.3389/fmicb.2020.01132)
Supplement: Supplementary file 3 [file Presentation_3.PPTX]

## Slide 1
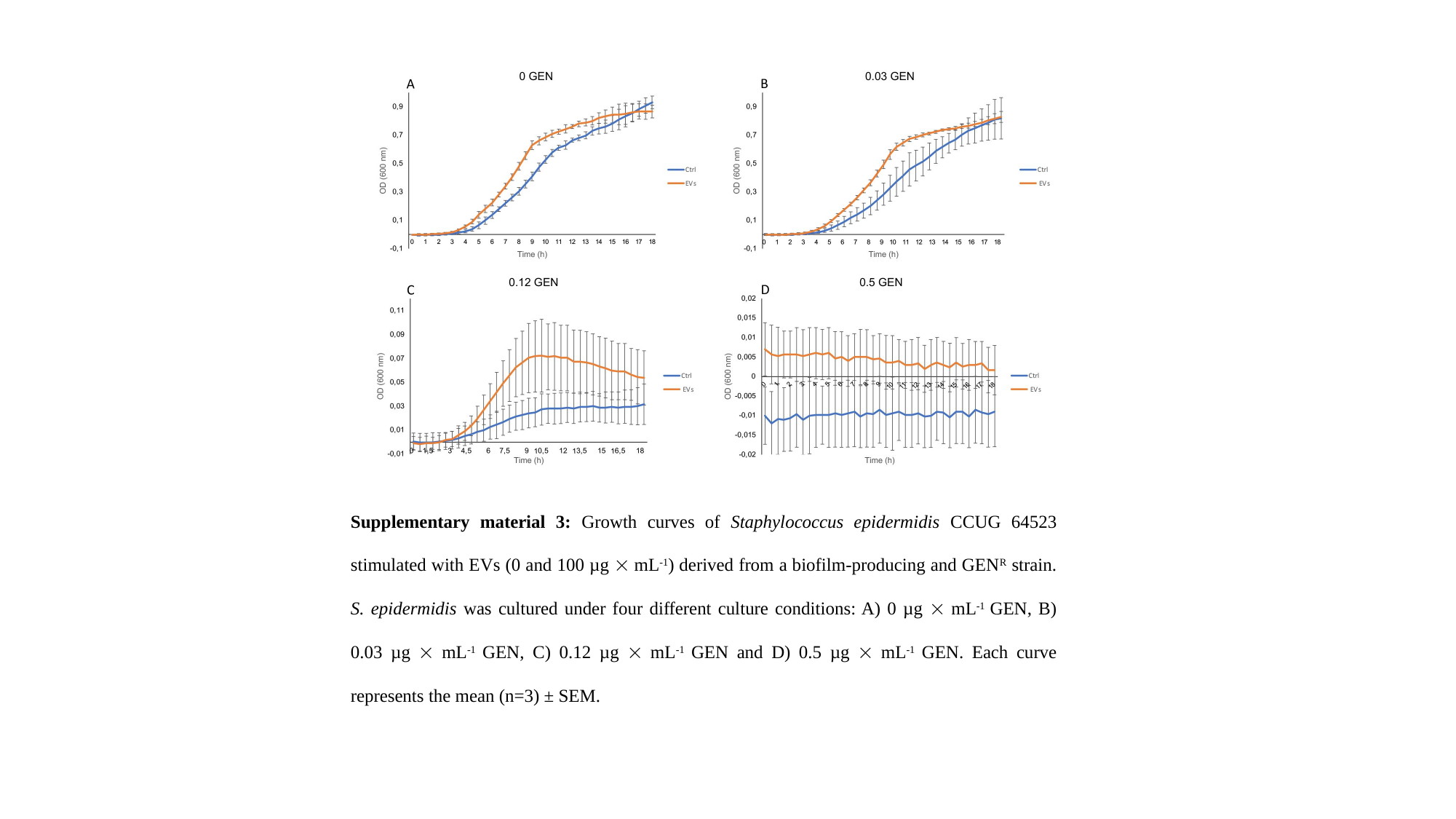

Supplementary material 3: Growth curves of Staphylococcus epidermidis CCUG 64523 stimulated with EVs (0 and 100 µg × mL-1) derived from a biofilm-producing and GENR strain. S. epidermidis was cultured under four different culture conditions: A) 0 µg × mL-1 GEN, B) 0.03 µg × mL-1 GEN, C) 0.12 µg × mL-1 GEN and D) 0.5 µg × mL-1 GEN. Each curve represents the mean (n=3) ± SEM.
